# Highlights in Applied Evolutionary Biology

**DOI:** 10.1111/eva.12053

**Published:** 2013-02-18

Below we highlight some recent papers that use evolutionary biology to address questions of applied importance.

## Fisheries

Sutter, D. A. H., C. D. Suski, D. P. Philipp, T. Klefoth, D. H. Wahl, P. Kersten, S. J. Cooke, and R. Arlinghaus. 2012 Recreational Fishing Selectively Captures Individuals with the Highest Fitness Potential. Proceedings of the National Academy of Science*s* 109, no. 51: 20960–20965.doi:10.1073/pnas.1212536109.

Fisheries-induced evolution and its impact on the productivity of exploited fish stocks remains a highly contested research topic in applied fish evolution and fisheries science. Although many quantitative models assume that larger, more fecund fish are preferentially removed by fishing, there is no empirical evidence describing the relationship between vulnerability to capture and individual reproductive fitness in the wild. Using males from two lines of largemouth bass (*Micropterus salmoides*) selectively bred over three generations for either high (HV) or low (LV) vulnerability to angling as a model system, we show that the trait “vulnerability to angling” positively correlates with aggression, intensity of parental care, and reproductive fitness. Our study constitutes experimental evidence that recreational angling selectively captures individuals with the highest potential for reproductive fitness. Our study further suggests that selective removal of the fittest individuals likely occurs in many fisheries that target species engaged in parental care. As a result, depending on the ecological context, angling-induced selection may have negative consequences for recruitment within wild populations of largemouth bass and possibly other exploited species in which behavioral patterns that determine fitness, such as aggression or parental care, also affect their vulnerability to fishing gear.

## Disease biology

Cornego et al. Evolutionary and population genomics of the cavity causing bacteria *Steptococcus mutans*.Molecular Biology and Evolution. *doi:10.1093/molbev/mss278*

*Streptococcus mutans* is widely recognized as one of the key etiological agents of human dental caries. Despite its role in this important disease our present knowledge of gene content variability across the species and its relationship to adaptation is minimal. Estimates of its demographic history are not available. In this study, we generated genome sequences of 57 *S. mutans* isolates, as well as representative strains of the most closely related species to *S. mutans* (*Streptococcus ratti, Streptococcus macaccae, Streptococcus criceti*), to identify the overall structure and potential adaptive features of the dispensable and core components of the genome. We also performed population genetic analyses on the core genome of the species aimed at understanding the demographic history, and impact of selection shaping its genetic variation. The maximum gene content divergence among strains was approximately 23%, with the majority of strains diverging by 5% to 15%. The core genome consisted of 1490 genes and the pan-genome approximately 3296. Maximum likelihood analysis of the synonymous site frequency spectrum (SFS) suggested that the *S. mutans* population started expanding exponentially around 10,000 years ago (95% CI: 3,268 — 14,344 ya), approximately coincidental with the onset of human agriculture. Analysis of the replacement SFS indicated that a majority of these substitutions are under strong negative selection, and the remainder evolved neutrally. A set of 14 genes was identified as being under positive selection, most of which were involved in either sugar metabolism or acid tolerance. Analysis of the core genome suggested that among 73 genes present in all isolates of *S. mutans* but absent in other species of the mutans taxonomic group, the majority can be associated with metabolic processes that could have contributed to the successful adaptation of *S. mutans* to its new niche, the human mouth, and with the dietary changes that accompanied the origin of agriculture.

Joseph, S. J., X. Didelot, J. Rothschild, H. J. C. de Vries, S. A. Morré, T. D. Read, and D. Dean. Population Genomics of Chlamydia Trachomatis: Insights on Drift, Selection, Recombination, and Population Structure. 2012Molecular Biology and Evolution. http://mbe.oxfordjournals.org/content/early/2012/08/31/molbev.mss198.short.

The large number of sexually transmitted diseases and ocular trachoma cases that are caused globally each year by *Chlamydia trachomatis* has made this organism a World Health Organization priority for vaccine development. However, there is no gene transfer system for *Chlamydia* to help identify potential vaccine targets. To accelerate discoveries toward this goal, here we analyzed the broadest diversity of *C. trachomatis* genomes to date, including 25 geographically dispersed clinical and seven reference strains representing 14 of the 19 known serotypes. Strikingly, all 32 genomes were found to have evidence of DNA acquisition by homologous recombination in their history. Four distinct clades were identified, which correspond to all *C. trachomatis* disease phenotypes: lymphogranuloma venereum (LGV; Clade 1); noninvasive urogenital infections (Clade 2); ocular trachoma (Clade 3); and protocolitis (Clade 4; also includes some noninvasive urogenital infections). Although the ancestral relationship between clades varied, most strains acted as donor and recipient of recombination with no evidence for barriers to genetic exchange. The niche-specific LGV and trachoma clades have undergone less recombination, although the opportunity for mixing with strains from other clades that infect the rectal and ocular mucosa, respectively, is evident. Furthermore, there are numerous occasions for gene conversion events through sequential infections at the same anatomic sites. The size of recombinant segments is relatively small (∼357 bp) compared with in vitro experiments of various *C. trachomatis* strains but is consistent with in vitro estimates for other bacterial species including *Escherichia coli* and *Helicobacter pylori*. Selection has also played a crucial role during the diversification of the organism. Clade 2 had the lowest nonsynonymous to synonymous ratio (d*N*/d*S*) but the highest effect of recombination, which is consistent with the widespread occurrence of synonymous substitutions in recombined genomic segments. The trachoma Clade 3 had the highest d*N*/d*S* estimates, which may be caused by an increased effect of genetic drift from niche specialization and a reduced effective population size. The degree of drift, selection, and recombination in *C. trachomatis* suggests that the challenge will remain to identify genomic regions that are stable and cross protective for the development of an efficacious vaccine.

Zhao, K., Y. Ishida, T. K. Oleksyk, C. A. Winkler and A. L. Roca. 2012. Evidence for selection at HIV host susceptibility genes in a West Central African human population BMC Evolutionary Biology 2012 12:237. http://www.biomedcentral.com/1471-2148/12/237

HIV-1 derives from multiple independent transfers of simian immunodeficiency virus (SIV) strains from chimpanzees to human populations. We hypothesized that human populations in west central Africa may have been exposed to SIV prior to the pandemic, and that previous outbreaks may have selected for genetic resistance to immunodeficiency viruses. To test this hypothesis, we examined the genomes of Biaka Western Pygmies, who historically resided in communities within the geographic range of the central African chimpanzee subspecies (Pan troglodytes troglodytes) that carries strains of SIV ancestral to HIV-1.

We found protective alleles or evidence for selection in the Biaka at a number of genes associated with HIV-1 infection or progression. Pygmies have also been reported to carry genotypes protective against HIV-1 for the genes CCR5 and CCL3L1. Our hypothesis that HIV-1 may have shaped the genomes of some human populations in West Central Africa appears to merit further investigation.

Kause, Antti, and JørgenØdegård. 2012. The Genetic Analysis of Tolerance to Infections: a Review. Frontiers in Genetics 3:252. doi:10.3389/fgene.2012.00262.

Tolerance to infections is defined as the ability of a host to limit the impact of a given pathogen burden on host performance. Uncoupling resistance and tolerance is a challenge, and there is a need to be able to separate them using specific trait recording or statistical methods. We present three statistical methods that can be used to investigate genetics of tolerance-related traits. Firstly, using random regressions, tolerance can be analyzed as a reaction norm slope in which host performance (y-axis) is regressed against an increasing pathogen burden (x-axis). Genetic variance in tolerance slopes is the genetic variance for tolerance. Variation in tolerance can induce genotype re-ranking and changes in genetic and phenotypic variation in host performance along the pathogen burden trajectory, contributing to environment-dependent genetic responses to selection. Such genotype-by-environment interactions can be quantified by combining random regressions and covariance functions. To apply random regressions, pathogen burden of individuals needs to be recorded. Secondly, when pathogen burden is not recorded, the cure model for time-until-death data allows separating two traits, susceptibility, and endurance. Susceptibility is whether or not an individual was susceptible to an infection, whereas endurance denotes how long time it took until the infection killed a susceptible animal (influenced by tolerance). Thirdly, the normal mixture model can be used to classify continuously distributed host performance, such as growth rate, into different sub-classes (e.g., noninfected and infected), which allows estimation of host performance reduction specific to infected individuals. Moreover, genetics of host performance can be analyzed separately in healthy and affected animals, even in the absence of pathogen burden and survival data. These methods provide novel tools to increase our understanding on the impact of parasites, pathogens, and production diseases on host traits.

## Conservation

Attard, C. R. M., Beheregaray, L. B., Jenner, K. C. S., Gill, P. C., Jenner, M.-N., Morrice, M. G., Robertson, K. M. and Möller, L. M. 2012. Hybridization of Southern Hemisphere blue whale subspecies and a sympatric area off Antarctica: impacts of whaling or climate change? Molecular Ecology, 21: 5715–5727.doi: 10.1111/mec.12025

Understanding the degree of genetic exchange between subspecies and populations is vital for the appropriate management of endangered species. Blue whales (*Balaenoptera musculus*) have two recognized Southern Hemisphere subspecies that show differences in geographic distribution, morphology, vocalizations, and genetics. During the austral summer feeding season, the Antarctic blue whale (*B. m. intermedia*) is found in polar waters and the pygmy blue whale (*B. m. brevicauda*) in temperate waters. Here, we genetically analyzed samples collected during the feeding season to report on several cases of hybridization between the two recognized blue whale Southern Hemisphere subspecies in a previously unconfirmed sympatric area off Antarctica. This means the pygmy blue whales using waters off Antarctica may migrate and then breed during the austral winter with the Antarctic subspecies. Alternatively, the subspecies may interbreed off Antarctica outside the expected austral winter breeding season. The genetically estimated recent migration rates from the pygmy to Antarctic subspecies were greater than estimates of evolutionary migration rates and previous estimates based on morphology of whaling catches. This discrepancy may be due to differences in the methods or an increase in the proportion of pygmy blue whales off Antarctica within the last four decades. Potential causes for the latter are whaling, anthropogenic climate change or a combination of these and may have led to hybridization between the subspecies. Our findings challenge the current knowledge about the breeding behavior of the world's largest animal and provide key information that can be incorporated into management and conservation practices for this endangered species.

## Climate change

Pauls, S. U., Nowak, C.,Bálint, M. and Pfenninger, M. 2012.The impact of global climate change on genetic diversity within populations and species. Molecular Ecology. doi: 10.1111/mec.12152

Genetic diversity provides the basic substrate for evolution, yet few studies assess the impacts of global climate change (GCC) on intraspecific genetic variation. In this review, we highlight the importance of incorporating neutral and nonneutral genetic diversity when assessing the impacts of GCC, for example, in studies that aim to predict the future distribution and fate of a species or ecological community. Specifically, we address the following questions: Why study the effects of GCC on intraspecific genetic diversity? How does GCC affect genetic diversity? How is the effect of GCC on genetic diversity currently studied? Where is potential for future research? For each of these questions, we provide a general background and highlight case studies across the animal, plant, and microbial kingdoms. We further discuss how cryptic diversity can affect GCC assessments, how genetic diversity can be integrated into studies that aim to predict species' responses on GCC and how conservation efforts related to GCC can incorporate and profit from inclusion of genetic diversity assessments. We argue that studying the fate of intraspecific genetic diversity is an indispensable and logical venture if we are to fully understand the consequences of GCC on biodiversity on all levels.

Tatters, A. O., Schnetzer, A., Fu, F., Lie, A. Y.A., Caron, D. A. and Hutchins, D. A. 2013. Short- versus long-term responses to changing CO_2_ in a coastal dinoflagellate bloom: implications for interspecific competitive interactions and community structure. Evolution. doi: 10.1111/evo.12029

Increasing *p*CO_2_ (partial pressure of CO_2_) in an “acidified” ocean will affect phytoplankton community structure, but manipulation experiments with assemblages briefly acclimated to simulated future conditions may not accurately predict the long-term evolutionary shifts that could affect inter-specific competitive success. We assessed community structure changes in a natural mixed dinoflagellate bloom incubated at three *p*CO_2_ levels (230, 433, and 765 ppm) in a short-term experiment (2 weeks). The four dominant species were then isolated from each treatment into clonal cultures, and maintained at all three *p*CO_2_ levels for approximately 1 year. Periodically (4, 8, and 12 months), these *p*CO_2_-conditioned clones were recombined into artificial communities, and allowed to compete at their conditioning *p*CO_2_ level or at higher and lower levels. The dominant species in these artificial communities of CO_2_-conditioned clones differed from those in the original short-term experiment, but individual species relative abundance trends across *p*CO_2_ treatments were often similar. Specific growth rates showed no strong evidence for fitness increases attributable to conditioning *p*CO_2_ level. Although *p*CO_2_ significantly structured our experimental communities, conditioning time and biotic interactions like mixotrophy also had major roles in determining competitive outcomes. New methods of carrying out extended mixed species experiments are needed to accurately predict future long-term phytoplankton community responses to changing *p*CO_2_.

## Invasion biology

Hodgins, K. A., Lai, Z., Nurkowski, K., Huang, J. and Rieseberg, L. H. 2013. The molecular basis of invasiveness: differences in gene expression of native and introduced common ragweed (*Ambrosia artemisiifolia*) in stressful and benign environments. Molecular Ecology. doi: 10.1111/mec.12179

Although the evolutionary and ecological processes that contribute to plant invasion have been the focus of much research, investigation into the molecular basis of invasion is just beginning. Common ragweed (*Ambrosia artemisiifolia*) is an annual weed native to North America and has been introduced to Europe where it has become invasive. Using a custom-designed NimbleGen oligoarray, we examined differences in gene expression between five native and six introduced populations of common ragweed in three different environments (control, light stress, and nutrient stress), as well as two different time points. We identified candidate genes that may contribute to invasiveness in common ragweed based on differences in expression between native and introduced populations from Europe. Specifically, we found 180 genes where range explained a significant proportion of the variation in gene expression and a further 103 genes with a significant range by treatment interaction. Several of these genes are potentially involved in the metabolism of secondary compounds, stress response and the detoxification of xenobiotics. Previously, we found more rapid growth and greater reproductive success in introduced populations, particularly in benign and competitive (light stress) environments, and many of these candidate genes potentially underlie these growth differences. We also found expression differences among populations within each range, reflecting either local adaptation or neutral processes, although no associations with climate or latitude were identified. These data provide a first step in identifying genes that are involved with introduction success in an aggressive annual weed.

Novy, A., Flory, S.L. and Hartman, J. M. 2012 Evidence for rapid evolution of phenology in an invasive grass. Journal of Evolutionary Biology.doi: 10.1111/jeb.12047

Evolutionary dynamics of integrative traits such as phenology are predicted to be critically important to range expansion and invasion success, yet there are few empirical examples of such phenomena. In this study, we used multiple common gardens to examine the evolutionary significance of latitudinal variation in phenology of a widespread invasive species, the Asian short-day flowering annual grass *Microstegium vimineum*. In environmentally controlled growth chambers, we grew plants from seeds collected from multiple latitudes across the species' invasive range. Flowering time and biomass were both strongly correlated with the latitude of population origin such that populations collected from more northern latitudes flowered significantly earlier and at lower biomass than populations from southern locations. We suggest that this pattern may be the result of rapid adaptive evolution of phenology over a period of less than 100 years and such changes have likely promoted the northward range expansion of this species. We note that possible barriers to gene flow, including bottlenecks and inbreeding, have apparently not forestalled evolutionary processes for this plant. Furthermore, we hypothesize that evolution of phenology may be a widespread and potentially essential process during range expansion for many invasive plant species.

